# Association between platelet count and 30-day in-hospital mortality among intensive care unit patients with sepsis: a multicenter retrospective cohort study

**DOI:** 10.3389/fmed.2024.1444481

**Published:** 2025-01-20

**Authors:** Jun Wang, Pan Zhou, Xin Li, Li Zhou, Zhe Deng

**Affiliations:** ^1^Department of Intensive Care Unit, Shenzhen Baoan Shi Yan People’s Hospital, Shenzhen, China; ^2^Department of Emergency Medicine, The First Affiliated Hospital of Shenzhen University, Shenzhen Second People’s Hospital, Shenzhen, China

**Keywords:** platelet, sepsis, 30-day in-hospital mortality, nonlinear, multicenter study

## Abstract

**Background:**

The relationship between platelet count and sepsis outcomes in intensive care units (ICUs) requires comprehensive investigation through large-scale multicenter studies.

**Methods:**

In this multicenter retrospective cohort study, we analyzed 17,977 sepsis patients from 208 U.S. hospitals (2014–2015) using the eICU Collaborative Research Database v2.0. Analyses were adjusted for demographics, clinical parameters, comorbidities, and treatments. Generalized additive models and two-piecewise linear regression were used to assess the relationship between platelet count and mortality.

**Results:**

A U-shaped relationship was identified with an inflection point at 176 × 10⁹/L. Below this threshold, each 10 × 10⁹/L increase in platelet count was associated with a 6% decrease in mortality risk (adjusted OR 0.94, 95% CI 0.93–0.95, *p* < 0.0001), while above it, each 10 × 10⁹/L increase was associated with a 1% increase in mortality risk (adjusted OR 1.01, 95% CI 1.00–1.01, *p* = 0.0153).

**Conclusion:**

This large-scale, multicenter retrospective study has made a significant contribution to understanding the association between platelet count and mortality in patients with sepsis in intensive care units. We identified a critical threshold of 176 × 10^9^/L for platelet count and demonstrated a distinct U-shaped relationship with 30-day in-hospital mortality, providing valuable reference criteria for clinical risk stratification.

## 1 Introduction

Sepsis, a dangerous dysfunction of the body’s organs, occurs when the body’s response to infection is not properly regulated (1). Annually, sepsis presents a substantial medical challenge, contributing to over 6 million deaths worldwide (2–4). In the United States, with a high incidence rate of 535 cases per 100,000 people annually and a hospital mortality rate of up to 30% (5, 6). As a result, the World Health Organization identifies it as a significant issue for public health (7). It is crucial to identify prognostic risk factors early in sepsis patients in order to enhance outcomes.

Platelet count is routine laboratory indicators, with their main functions being hemostasis and coagulation (8). Recent research links platelet not just to thrombosis but also other physiological and pathological processes. Research links platelet count and mean platelet volume with tumor metastasis (9, 10). Furthermore, research with 2056 intensive care unit (ICU) patients revealed a correlation between thrombocytopenia, acute respiratory distress syndrome, and mortality (11). A study involving 84 critically ill patients found that increased platelet volume following admission to the ICU is linked to a higher risk of in-hospital mortality (12). Studies examining either platelet count or platelet volume consistently indicate a possible link between platelets and patient outcomes.

Notably, a single-center retrospective study demonstrated an increased 28-day mortality rate among ICU sepsis patients with thrombocytopenia (13). Meanwhile, in septic patients, platelets undergo significant functional and quantitative changes, affecting both immune response and coagulation pathways (14, 15). However, there is a lack of multicenter large-scale research on the correlation between platelets and the outcome of sepsis. Using top-notch data from multicenter and extensive samples can offer dependable proof for future studies in this field.

Hence, we analyzed the eICU-CRD v2.0 data to explore the connection between initial platelet levels and the likelihood of 30-day in-hospital death in septic ICU patients.

## 2 Methods

### 2.1 Study design

In this retrospective cohort study conducted at multiple centers, the focus is on the relationship between initial platelet count and 30-day in-hospital mortality among sepsis patients, categorized as either deceased or alive.

### 2.2 Data source

The research made use of information from the eICU-CRDv2.0 database, which is a resource that is accessible to the public and was created through a partnership between Philips Healthcare and the Laboratory of Computational Physiology (LCP) at the Massachusetts Institute of Technology (MIT). The database holds vast information on 200,859 patients who were hospitalized in ICUs in 208 U.S. hospitals between 2014 and 2015. It covers a variety of clinical data, such as important measurements, nursing strategies, illness seriousness, diagnoses, and specifics of treatment (16). Registration is necessary to access the data, and the data collection process follows the guidelines established by the MIT Ethics Committee in accordance with the principles outlined in the 1964 Declaration of Helsinki. The author of this article, Pan Zhou, obtained access to the database and conducted the data collection.

### 2.3 Study population

**Figure 1:**
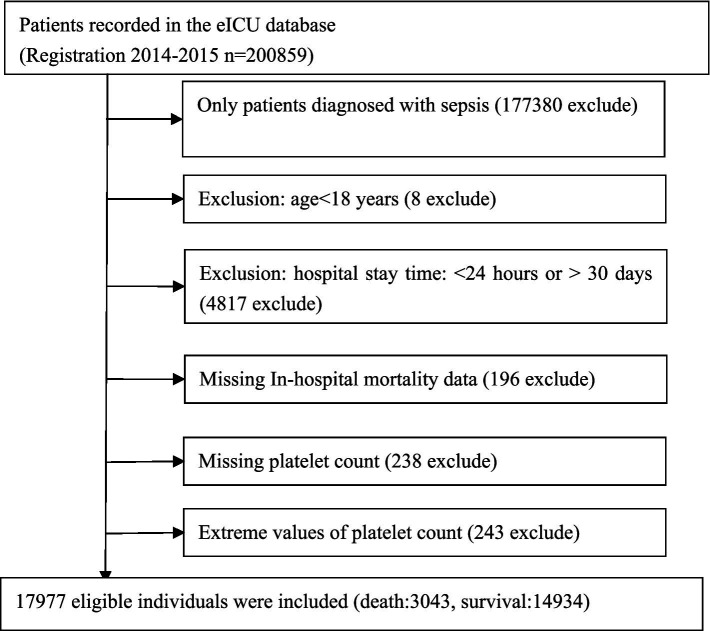
Flowchart of study participants.

The research involved 17,977 qualified participants after implementing various exclusion criteria, such as excluding individuals who were not sepsis patients, those under 18 years old, those with ICU stays shorter than 24 h or longer than 30 days, those with missing in-hospital mortality data, those with missing platelet count, and those with extremely high or low (three standard deviations above or below the mean) platelet counts. Figure 1 illustrates the process of selecting participants.

### 2.4 Variables

Platelet counts were initially recorded as continuous values. Based on established clinical reference ranges (8, 17), we classified patients into three groups according to their platelet counts: below normal (<150 × 10^9^/L), normal range (150–400 × 10^9^/L), and above normal (>400 × 10^9^/L).

### 2.5 Covariates

The covariates were selected based on clinical experience and findings from previous studies (13, 18–22). Variable such as gender, race, acute respiratory failure (ARF), atrial fibrillation (AF), acute coronary syndrome (ACS), congestive heart failure (CHF), chronic obstructive pulmonary disease (COPD), stroke, diabetes, hypertension, antiplatelet medication, glucocorticoid medication, carbapenem medication, cephalosporin medication, levofloxacin medication, vancomycin medication, and use of mechanical ventilation are considered categorical. Age, hemoglobin concentration (Hb), blood urea nitrogen (BUN), serum creatinine (Scr), albumin (ALB), and Acute Physiology and Chronic Health Evaluation-IV score (APACHE-IV score) are all continuous variables. Initial parameters were assessed within the first 24 h of ICU admission.

### 2.6 Missing data processing

Missing data analysis revealed varying degrees of missingness across variables: hemoglobin (Hb, 0.01%, *n* = 2), blood urea nitrogen (BUN, 0.13%, *n* = 24), serum creatinine (Scr, 0.19%, *n* = 35), albumin (ALB, 7.73%, *n* = 1,389), and APACHE-IV scores (13.55%, *n* = 2,435). Analysis of missing patterns showed that most variables had less than 1% missing values, except for ALB and APACHE-IV scores. Little’s MCAR test indicated that data were missing at random (MAR) (23). We performed multiple imputation using the Markov Chain Monte Carlo method with chained equations, generating 5 imputed datasets. The imputation model included all relevant demographic and clinical variables to ensure robust estimation. To validate our approach, we conducted sensitivity analyses comparing results between the imputed datasets and complete-case analysis. Additional sensitivity analyses using alternative imputation methods (predictive mean matching and random forest) demonstrated consistent results across all approaches, with primary associations remaining statistically significant. Given the low missingness in key outcome variables and the consistency of results across different analytical approaches, we concluded that the potential impact of missing data on our findings was minimal.

### 2.7 Statistical analysis

Individuals were divided into four categories according to their platelet levels. Continuous variables were presented as the mean with standard deviation for normally distributed data and as the median with interquartile range for non-normally distributed data. Categorical variables were presented as numbers and percentages. We utilized one-way analysis of variance for normally distributed data, the χ2 test for categorical variables, and the Kruskal-Wallis H test to examine variations among platelet count groups.

#### 2.7.1 To examine the autonomous linear correlation between platelet levels and mortality within 30 days of hospitalization

The importance of a 1 × 10^9^/L increase or decrease in platelet count is thought to be minimal in clinical terms. Hence, when conducting multivariate regression analysis, it is more beneficial to divide the platelet count by 10 for increased clinical relevance. The authors initially conducted collinearity screening (see Supplementary Table S1, where resulted no covariates being excluded). Three models were built by them using multivariate binary logistic regression, in accordance with the STROBE statement (24), in order to investigate the relationship between platelet count and 30-day in-hospital mortality. The models included the following: (1) basic model (no covariates adjusted); (2) partially adjusted model (Model I adjusted for gender, age, and race); (3) fully adjusted model (Model II adjusted for age, gender, race, Hb, platelet, BUN, Scr, ALB, APACHE-IV score, ARF, AF, ACS, CHF, COPD, stroke, diabetes mellitus, hypertension, antiplatelet, glucocorticoids, carbapenems, cephalosporins, levofloxacin, vancomycin, and mechanical ventilation). Measurements of effect sizes were documented along with their corresponding 95% confidence intervals (95% CIs). Covariates were adjusted in the model if their addition caused a 10% or greater change in the odds ratio (OR) (24). Furthermore, the possibility of unmeasured confounding between platelet count and 30-day in-hospital mortality was assessed through the use of E-values.

#### 2.7.2 Interaction and subgroup analyses

Interaction and subgroup analyses were performed to evaluate the strength of the findings. A stratified binary logistic regression model was utilized by the researchers to conduct interaction and subgroup analyses across a range of subgroups, including gender, race, ARF, AF, ACS, CHF, COPD, stroke, diabetes, hypertension, and mechanical ventilation. Besides the stratification factor, every stratum was modified for various variables such as age, gender, race, Hb, platelet count, BUN, Scr, ALB, APACHE-IV scores, ARF, AF, ACS, CHF, COPD, stroke, diabetes, hypertension, antiplatelet, glucocorticoids, carbapenems, cephalosporins, vancomycin, levofloxacin, and mechanical ventilation. Interaction tests were performed by employing the likelihood ratio test to assess the difference between models with and without interaction terms (25, 26).

#### 2.7.3 Examining the non-linear correlation between platelet levels and mortality within 30 days of hospitalization

Linear relationships sometimes fail to reflect the true relationship between two variables. In order to account for possible nonlinearity, the researchers utilized generalized additive models (GAM) and applied smooth curve fitting (penalized spline method) to further explore the nonlinear association between platelet count and 30-day in-hospital mortality. The Generalized Additive Model (GAM) is essentially a flexible statistical modeling approach that enables the exploration of nonlinear relationships between predictor variables (such as blood pressure and age) and outcome variables (such as mortality and complications). The smooth curve fitting (penalized spline method) is fundamentally a mathematical approach that demonstrates relationships between variables by fitting smooth curves through data points. The optimal degrees of freedom for the smooth terms were selected using the generalized cross-validation criterion. The recursive algorithm used to identify the inflection point involved iteratively fitting two-piece linear regression models at different potential threshold values and selecting the point that maximized the likelihood function. In the presence of nonlinearity, our approach involved initially identifying the inflection point using a recursive algorithm. Following that, we developed a binary logistic regression model with two components on each side of the inflection point (27). The two-piecewise linear regression model was then compared with the standard linear model using likelihood ratio tests to confirm the superior fit of the nonlinear approach (*p* < 0.001).

All results adhered to the STROBE statement (24). Analyses were conducted using the statistical software packages R (R Foundation)^2^ and EmpowerStats^3^ (X&Y Solutions, Inc., Boston, MA). Significant results were identified with a *p*-value less than 0.05 (two-tailed) according to statistical analysis.

## 3 Results

### 3.1 Characteristics of individuals

**Figure 2:**
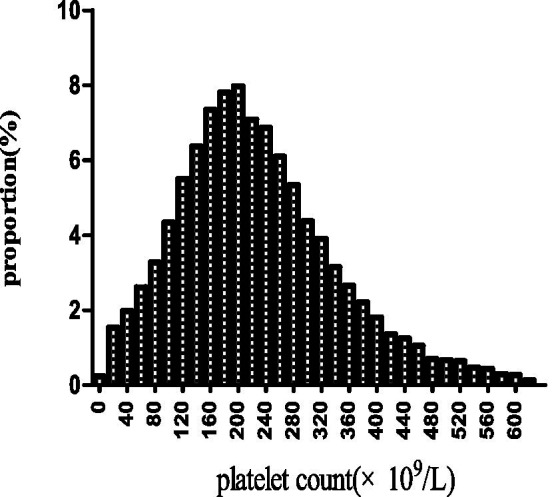
The spread of platelet levels. It exhibits approximate normal distribution of platelet count, with an average of 226 × 10^9^/l and a standard deviation of 114 × 10^9^/l.

Figure 2 illustrates the approximate normal distribution of platelet count, with an average of 226 × 10^9^/l and a standard deviation of 114 × 10^9^/l. Table 1 presents the baseline characteristics of 17,977 participants, categorized by platelet levels (Q1-Q3). Significant differences were observed in demographic characteristics across groups, with male predominance gradually decreasing from Q1 to Q3 (59.04, 50.33, and 46.83%, respectively; *p* < 0.001). The ethnic distribution showed slight variations (*p* = 0.006), with Caucasians comprising the majority in all groups (74.90–77.53%). Clinical parameters demonstrated significant inter-group differences. The Q1 group exhibited the highest APACHE-IV scores (74.94 ± 28.15 vs. 68.81 ± 26.34 and 69.63 ± 25.99 in Q2 and Q3, respectively; *p* < 0.001) and elevated renal function markers (median BUN: 30.00 vs. 26.00 mg/dL; median SCr: 1.50 vs. 1.29 and 1.15 mg/dL; all *p* < 0.001). ALB levels were highest in the Q2 group (2.93 ± 0.74 g/dL; *p* < 0.001). Regarding comorbidities, significant differences were noted in the prevalence of AF (14.56, 14.47, and 9.99% for Q1, Q2, and Q3, respectively; p < 0.001), COPD (8.31, 11.38, and 10.05%; *p* < 0.001), and diabetes mellitus (13.83, 16.91, and 15.65%; p < 0.001). Treatment patterns were largely similar across groups, except for antiplatelet therapy, which was more frequently administered in the Q2 group (6.02% vs. 4.47 and 4.25%; *p* < 0.001). Notably, 30-day in-hospital mortality demonstrated a significant difference across groups (p < 0.001), with the highest rate observed in Q1 (22.76%), followed by Q3 (17.27%), while Q2 showed the lowest mortality rate (14.59%).

**Table 1:** Baseline characteristics of participants (*N* = 17,977).

Platelet (×10^9^/l)	Q1 (1–150)	Q2 (150–400)	Q3 (400–619)	*P*-value
Participants	4,658	11,837	1,482	
Sex				<0.001
Male	2,750 (59.04%)	5,957 (50.33%)	694 (46.83%)	
Female	1908 (40.96%)	5,880 (49.67%)	788 (53.17%)	
Ethnicity				0.006
Caucasian	3,489 (74.90%)	9,172 (77.49%)	1,149 (77.53%)	
African American	491 (10.54%)	1,160 (9.80%)	148 (9.99%)	
Hispanic	248 (5.32%)	540 (4.56%)	73 (4.93%)	
Asian	92 (1.98%)	262 (2.21%)	23 (1.55%)	
Other/unknown	338 (7.26%)	703 (5.94%)	89 (6.01%)	
Age (years)	64.76 (15.89)	65.71 (16.30)	63.21 (16.17)	<0.001
Hb (g/dl)	10.93 (2.59)	11.61 (2.49)	10.95 (2.55)	<0.001
BUN((mg/dl))	30.00 (18.00–48.00)	26.00 (16.00–43.00)	26.00 (15.00–44.00)	<0.001
Scr (mg/dl)	1.50 (0.96–2.60)	1.29 (0.86–2.20)	1.15 (0.76–2.00)	<0.001
ALB (g/dl)	2.71 (0.71)	2.93 (0.74)	2.74 (0.76)	<0.001
APACHE-IV score	74.94 (28.15)	68.81 (26.34)	69.63 (25.99)	<0.001
Comorbid conditions				
ARF	1,491 (32.01%)	3,986 (33.67%)	466 (31.44%)	0.048
AF	678 (14.56%)	1713 (14.47%)	148 (9.99%)	<0.001
ACS	339 (7.28%)	871 (7.36%)	86 (5.80%)	0.090
CHF	489 (10.50%)	1,356 (11.46%)	130 (8.77%)	0.004
COPD	387 (8.31%)	1,347 (11.38%)	149 (10.05%)	<0.001
Stroke	129 (2.77%)	372 (3.14%)	27 (1.82%)	0.013
Diabetes mellitus	644 (13.83%)	2002 (16.91%)	232 (15.65%)	<0.001
Hypertension	452 (9.70%)	1,382 (11.68%)	143 (9.65%)	<0.001
Treatment				
Antiplatelet	208 (4.47%)	713 (6.02%)	63 (4.25%)	<0.001
Glucocorticoid	507 (10.88%)	1,203 (10.16%)	147 (9.92%)	0.337
Carbapenems	199 (4.27%)	444 (3.75%)	71 (4.79%)	0.073
Cephalosporins	538 (11.55%)	1,303 (11.01%)	143 (9.65%)	0.125
Levofloxacin	328 (7.04%)	871 (7.36%)	104 (7.02%)	0.731
Vancomycin	884 (18.98%)	2,112 (17.84%)	277 (18.69%)	0.207
Mechanical ventilation	1,577 (33.86%)	4,067 (34.36%)	537 (36.23%)	0.243
Mortality	1,060 (22.76%)	1727 (14.59%)	256 (17.27%)	<0.001

Categorical information is presented as n (%). Continuous data is presented as mean ± SD or median (Q1-Q3). Hb, hemoglobin; BUN, blood urea nitrogen, Scr, creatinine; ALB, albumin; APACHE-IV scores, Acute Physiology and Chronic Health Evaluation-IV score; ARF, acute respiratory failure; AF, atrial fibrillation; ACS, acute coronary syndrome; CHF, congestive heart failure; COPD, chronic obstructive pulmonary disease; Mortality, 30 day in-hospital mortality.

### 3.2 Outcomes of mortality within 30 days of hospital admission

Table 2 shows the 30-day mortality rates among 17,977 sepsis patients. The total mortality rate within 30 days of hospital admission was 16.9% (95% CI: 16.4–17.4%). Analysis of mortality rates across platelet count categories revealed a non-linear relationship. The highest mortality rate was observed in the Q1 group (platelet count 1–150 × 109/L) at 22.8% (95% CI: 21.6–24.0%), while the Q2 group (platelet count 150–400 × 109/L) demonstrated the lowest mortality rate at 14.6% (95% CI: 14.0–15.2%). Patients in the Q3 group (platelet count 400–619 × 109/L) exhibited an intermediate mortality rate of 17.3% (95% CI: 15.4–19.2%). The non-overlapping confidence intervals among groups suggest statistically significant differences in mortality rates across these platelet count categories.

**Table 2:** The relationship between platelet levels and 30-day inpatient mortality in individual with sepsis.

Platelet	Participants (n)	Events	Mortality rate (%)	(95% CI)
Total	17,977	3,043	16.9	16.4–17.4
Q1	4,658	1,060	22.8	21.6–24.0
Q2	11,837	1727	14.6	14.0–15.2
Q3	1,482	256	17.3	15.4–19.2

### 3.3 Result of univariate analyses utilizing a binary logistic regression model

Supplementary Table S2 summarizes the results of the univariate regression analysis conducted to the factors linked to 30-day in-hospital mortality. Older age was associated with higher chances of 30-day inpatient death(OR 1.02, 95% CI 1.02–1.02, *p* < 0.0001). Elevated platelet count (OR: 0.98, 95% CI: 0.98–0.99, *p* < 0.0001), Hb (OR 0.93, 95% CI 0.92–0.95, *p* < 0.0001), and ALB (OR 0.54, 95% CI 0.51–0.57, *p* < 0.0001) levels were correlated with decreased odds of mortality. On the other hand, higher BUN levels(OR 1.01, 95% CI 1.01–1.01, *p* < 0.0001), Scr (OR 1.04, 95% CI 1.02–1.06, *p* < 0.0001), and APACHE-IV scores (OR 1.03, 95% CI 1.03–1.03, *p* < 0.0001) were associated with higher chances of death. The likelihood of 30-day in-hospital mortality was higher with the presence of ARF (OR 2.24, 95% CI 2.07–2.42, *p* < 0.0001), AF (OR 1.77, 95% CI 1.60–1.96, *p* < 0.0001), ACS (OR 1.71, 95% CI 1.50–1.95, *p* < 0.0001), CHF (OR 1.37, 95% CI 1.22–1.54, *p* < 0.0001), and stroke (OR 2.00, 95% CI 1.65–1.42, *p* < 0.0001) among concurrent medical conditions. In contrast, mortality was not significantyl linked to COPD, diabetes mellitus, and hypertension. Regarding treatment factors, the use of glucocorticoids (OR 1.21, 95% CI 1.07–1.37, *p* = 0.0023), carbapenems (OR 1.33, 95% CI 1.11–1.60, *p* = 0.0022), and vancomycin (OR 1.20, 95% CI 1.09–1.33, *p* = 0.0002) were associated with higher odds of mortality, while the use of cephalosporins was associated with lower odds (OR 0.87, 95% CI 0.76–0.99, *p* = 0.0318). The use of mechanical ventilation signifcanty raised the chances of in-hospital mortality within 30 days(OR 2.93, 95% CI 2.70–3.17, *p* < 0.0001). Notably, the use of antiplatelet medication and Levofloxacin did not demonstrate significant associations with mortality.

### 3.4 Results of multivariate analyses utilizing the binary logistic regression model

The association between platelet counts and 30-day in-hospital mortality was examined using both continuous and categorical analyses across three progressive adjustment models (Table 3). In the continuous analysis, platelet count demonstrated a consistent protective effect, with each 10 × 109/L increase associated with a 2% reduction in mortality risk in both the crude model and Model I (OR = 0.98, 95% CI: 0.98–0.99, *p* < 0.0001). This protective effect persisted, though slightly attenuated, in the fully adjusted Model II (OR = 0.99, 95% CI: 0.98–0.99, *p* < 0.0001).

**Table 3:** Correlation between platelet levels and the rate of in-hospital mortality within 30 days across various models.

Exposure	Crude model (OR, 95%CI, *P*)	Model I (OR, 95%CI, *P*)	Model II (OR, 95%CI, *P*)
Platelet/10	0.98 (0.98, 0.99) <0.0001	0.98 (0.98, 0.99) <0.0001	0.99 (0.98, 0.99) <0.0001
Platelet(×10^9^/l)			
Q2 (150–400)	Ref.	Ref.	Ref.
Q3 (400–619)	1.22 (1.06, 1.41) 0.0063	1.29 (1.12, 1.50) 0.0005	1.20 (1.02, 1.40) 0.0238
Q1 (1–150)	1.72 (1.58, 1.88) <0.0001	1.77 (1.63, 1.93) <0.0001	1.57 (1.43, 1.73) <0.0001

CI, confidence interval. Model I modified to account for age, gender, and race. Model II modified to account for age, gender, race, Hb, BUN, Scr, ALB, APACHE-IV score, ARF, AF, ACS, CHF, COPD, stroke, diabetes mellitus, hypertension, antiplatelet, glucocorticoid, carbapenems, cephalosporins, levofloxacin, vancomycin, and mechanical ventilation.

When analyzed as categorical variables using the normal platelet count range (150–400 × 109/L) as reference, both low and high platelet counts were associated with increased mortality risk, albeit to different degrees. Notably, thrombocytopenia (1–150 × 109/L) demonstrated the strongest association with mortality. In the crude model, patients with low platelet counts showed a 72% increased risk of death (OR = 1.72, 95% CI: 1.58–1.88, *p* < 0.0001), and this association remained robust after full adjustment (OR = 1.57, 95% CI: 1.43–1.73, *p* < 0.0001). Thrombocytosis (400–619 × 109/L) was associated with a more modest but still significant increase in mortality risk, with an adjusted odds ratio of 1.20 (95% CI: 1.02–1.40, *p* = 0.0238) in the fully adjusted model.

### 3.5 Interaction and subgroup analysis results

Table 4 shows how platelet levels relate to 30-day mortality in different patient subgroups. We identified two significant interactions: sex (P for interaction = 0.0178) and diabetes status (P for interaction = 0.0174). In males (52.3%), higher platelet counts were more strongly associated with lower mortality (OR = 0.98, 95% CI: 0.98–0.99, *p* < 0.0001) than in females (47.7%, OR = 0.99, 95% CI: 0.99–1.00, *p* = 0.0038). Similarly, non-diabetic patients (83.99%) showed a protective effect (OR = 0.99, 95% CI: 0.98–0.99, *p* < 0.0001), while diabetic patients (16.01%) showed no significant effect (OR = 1.00, 95% CI: 0.99–1.01, *p* = 0.7267). Other clinical factors, including ethnicity, acute respiratory failure, atrial fibrillation, acute coronary syndrome, congestive heart failure, COPD, stroke, hypertension, and mechanical ventilation showed no significant interactions (all P for interaction >0.05). The findings indicate that this relationship is robust and not significantly influenced by most demographic and clinical factors.

**Table 4:** Findings from the analysis of interaction analysis and subgroup.

Characteristic	No of participants	OR95%CI, P	P for interaction
Sex			0.0178
Male	9,401 (52.3%)	0.98 (0.98, 0.99) <0.0001	
Female	8,576 (47.7%)	0.99 (0.99, 1.00) 0.0038	
Ethnicity			0.5879
Caucasian	13,810 (76.82%)	0.99 (0.98, 0.99) <0.0001	
African American	1799 (10.01%)	0.99 (0.98, 1.00) 0.1329	
Hispanic	861 (4.79%)	0.98 (0.97, 1.00) 0.0754	
Asian	377 (2.10%)	1.00 (0.97, 1.03) 0.8662	
Other/unknown	1,130 (6.29%)	0.98 (0.96, 0.99) 0.0027	
ARF			0.3605
No	12,034 (66.94%)	0.99 (0.98, 0.99) <0.0001	
Yes	5,943 (33.06%)	0.99 (0.98, 1.00) 0.0003	
AF			0.6976
No	15,438 (85.88%)	0.99 (0.98, 0.99) <0.0001	
Yes	2,539 (14.12%)	0.99 (0.98, 1.00) 0.0225	
ACS			0.1489
No	16,681 (92.79%)	0.99 (0.98, 0.99) <0.0001	
Yes	1,296 (7.21%)	1.00 (0.98, 1.01) 0.5703	
CHF			0.9682
No	16,002 (89.01%)	0.99 (0.98, 0.99) <0.0001	
Yes	1975 (10.99%)	0.99 (0.98, 1.00) 0.0247	
COPD			0.2658
No	16,094 (89.53%)	0.99 (0.98, 0.99) <0.0001	
Yes	1883 (10.47%)	0.99 (0.98, 1.01) 0.3149	
Stroke			0.4685
No	17,449 (97.06%)	0.99 (0.98, 0.99) <0.0001	
Yes	528 (2.94%)	0.98 (0.96, 1.00) 0.0569	
Diabetes mellitus			0.0174
No	15,099 (83.99%)	0.99 (0.98, 0.99) <0.0001	
Yes	2,878 (16.01%)	1.00 (0.99, 1.01) 0.7267	
Hypertension			0.8827
No	16,000 (89.00%)	0.99 (0.98, 0.99) <0.0001	
Yes	1977 (11.00%)	0.99 (0.98, 1.00) 0.0696	
Mechanical ventilation			0.5154
No	11,796 (65.62%)	0.99 (0.98, 0.99) <0.0001	
Yes	6,181 (34.38%)	0.99 (0.98, 0.99) <0.0001	

ARF, acute respiratory failure; AF, atrial fibrillation; ACS, acute coronary syndrome; CHF, congestive heart failure; COPD, chronic obstructive pulmonary disease.

### 3.6 Addressing nonlinearity with GAM

**Figure 3:**
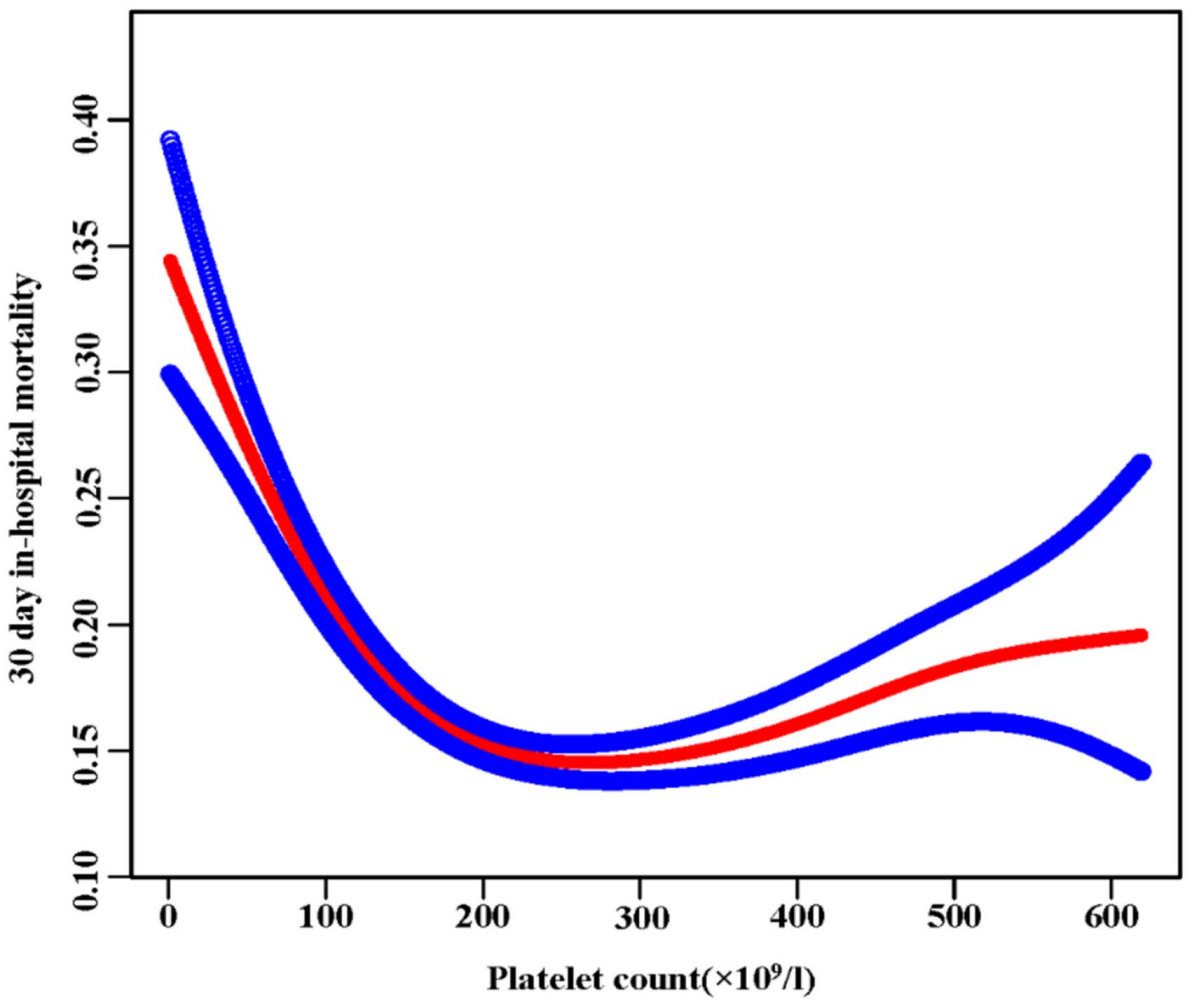
The nonlinear relationship between platelet and 30-day in-hospital mortality. A nonlinear relationship was detected after adjusting for age, sex, ethnicity, Hb, BUN, Scr, ALB, APACHE-IV score, ARF, AF, ACS, CHF, COPD, stroke, diabetes, hypertension, antiplatelet, glucocorticoid, carbapenems, cephalosporins, levofloxacin, vancomycin, and mechanical ventilation (Log-likelihood ratio test *p* < 0.001).

We found a nonlinear relationship between platelet count and mortality (Figure 3). Our analysis revealed a critical threshold at 176 × 109/L platelets. This relationship remained significant after adjusting for demographic and clinical factors (Log-likelihood ratio test *p* < 0.001) (Table 5). Below 176 × 109/L, each 10 × 109/L increase in platelets reduced mortality risk by 6% (OR 0.94, 95% CI 0.93–0.95, *p* < 0.0001). Above this threshold, each 10 × 109/L increase was associated with a slight 1% increase in mortality risk (OR 1.01, 95% CI 1.00–1.01, *p* = 0.0153). The two-piecewise regression model provided a better fit than the standard linear model (P for log-likelihood ratio test <0.001), confirming the nonlinear nature of the relationship between platelet count and mortality.

**Table 5:** The result of the two-segmented linear regression model.

30-day in-hospital mortality	OR, 95%CI, *P*	*P*-value
Fitting model by standard linear regression	0.99 (0.98, 0.99)	<0.0001
Fitting model by two-piecewise regression		
Inflection point of platelet count	176 × 10^9^/l	
≤Inflection point	0.94 (0.93, 0.95)	<0.0001
> Inflection point	1.01 (1.00, 1.01)	0.0153
P for log-likelihood ratio test		<0.001

## 4 Discussion

This multicenter study investigated how initial platelet counts relate to 30-day mortality in ICU patients with sepsis. We found a nonlinear relationship between platelet count and mortality, with a clear threshold effect. When platelet count falls below 176 × 10^9^/l, a rise of 10 × 10^9^/l in platelet count is linked to a 6% reduction in 30-day in-hospital mortality. On the other hand, when the platelet count goes over 176 × 10^9^/l, a 1% rise in the 30-day in-hospital mortality is linked to every 10 × 10^9^/l increase.

This study not only underscores the correlation between a higher initial platelet count and reduced mortality in ICU patients with sepsis but also reveals a saturation threshold effect. Initially, the researchers investigated the correlation between initial platelet levels and the risk of mortality within 30 days of hospitalization in septic patients in the ICU using both univariate and multivariate logistic regression methods. A greater platelet count showed a strong and independent relationship with decreased 30-day mortality (*p* < 0.0001). Furthermore, this relationship demonstrated consistent robustness across the majority of subgroup analyses. Additionally, using GAM and smooth curve fitting techniques, the authors investigated the nonlinear correlation between initial platelet count and 30-day in-hospital mortality among individuals with sepsis. In a medical setting, this discovery suggests that for individuals with a significant decrease in platelets, even slight improvements in platelet levels could result in substantial improvements in survival rates. However, beyond a platelet count of 176 × 109/L, additional elevations in platelet count are linked to a higher 30-day in-hospital mortality rate in ICU septic patients. In conclusion, the two-segmented regression models indicate a critical turning point in the relationship between platelet levels and death rates. This information could be of significantly importance for risk stratification among sepsis patients.

The association between initial platelet counts and outcomes in critically ill patients has attracted increasing research attention. Multiple studies have demonstrated that thrombocytopenia correlates with increased mortality across various critical illness populations (28–31). Notably, Wang et al. conducted a single-center study suggesting a nonlinear relationship between platelet count and ICU mortality in sepsis patients, with an inflection point at 100 × 10^9^/L (13). They observed inverse relationships between platelet counts and 28-day mortality on either side of this threshold. In the same way, our multicenter study similarly identified a nonlinear relationship between admission platelet counts and 30-day mortality in ICU sepsis patients, but with a higher inflection point of 176 × 109/L. This difference in threshold values likely reflects our larger, multicenter design and more comprehensive confounder adjustment, including E-value analysis to assess unmeasured confounding. Given that thrombocytopenia is conventionally defined as platelet counts below 150 × 10^9^/L (17). Our identified threshold of 176 × 10^9^/L may represent a more clinically relevant cutoff point for risk stratification.

The relationship between platelet count and sepsis mortality has been extensively studied but remains complex (32–34). Based on current evidence, we propose that below the identified threshold, low platelet counts correlate with increased mortality through four main mechanisms. First, reduced platelets impair hemostasis and coagulation, increasing the risk of potentially fatal bleeding in critical organs, increasing the risk of potentially fatal bleeding in critical organs (33–37). Second, thrombocytopenia often indicates disease severity (38–41). Our data show that patients in the lowest platelet quartile (Q1) had significantly higher BUN, Scr, and APACHE-IV scores, suggesting greater illness severity. Third, platelets play crucial roles in innate immunity (34, 42–45), including pathogen elimination and immune cell interactions. Therefore, low platelet counts may signal compromised immunity and increased mortality risk. Fourth, severe thrombocytopenia is characteristic of disseminated intravascular coagulation (DIC), a serious complication of sepsis that leads to systemic microvascular thrombosis and subsequent multi-organ failure (35, 45). Above the threshold, elevated platelet counts associate with increased mortality through three potential mechanisms. First, enhanced thrombotic tendency during systemic inflammatory responses may promote excessive thrombosis (46–48). Second, higher platelet counts may amplify inflammatory responses through increased platelet-pathogen interactions and subsequent release of pro-inflammatory mediators (34). Third, recent studies have shown that platelets can undergo pyroptosis during severe sepsis, a form of inflammatory cell death that combines features of apoptosis and necrosis, potentially exacerbating tissue damage and organ dysfunction (49). This complex relationship suggests that maintaining platelet counts within an optimal range, rather than simply preventing thrombocytopenia, might be crucial for improving outcomes in sepsis patients (34).

The research has multiple advantage that increase the trustworthiness of its results. First, the multi-center approach and the relatively large sample size improve the generalizability of the results. Second, employing multiple imputation for missing data helps minimize bias, ultimately increasing statistical power and strengthening the reliability of our results. Third, we conducted interaction and subgroup analyses to further validate the reliability of our results. Additionally, the calculation of the E-value indicated that other unmeasured confounding factors had minimal impact, further supporting the generalizability of our conclusions.

Several limitations of this observational study warrant consideration. First, our analysis was restricted to data from 208 US hospitals, potentially limiting the generalizability of findings to other geographical regions and populations. Second, the observational design precluded establishing causal relationships between platelet counts and sepsis outcomes, highlighting the need for prospective studies to investigate underlying mechanisms. Third, despite controlling for major confounders, some potential factors, particularly hematological diseases, were not included in our analysis due to data availability constraints. Fourth, the eICU-CRD v2.0 database lacks detailed sepsis severity classification, especially the distinction between sepsis and septic shock, which limited our ability to fully evaluate disease progression. Fifth, the absence of specific cause-of-death data prevented us from assessing cause-specific mortality, which could have provided additional insights into the relationship between platelet counts and specific fatal complications.

## 5 Conclusion

In conclusion, this study establishes a critical platelet count threshold (176 × 10^9^/L) in sepsis patients, demonstrating a U-shaped relationship with 30-day mortality. These findings provide valuable reference points for clinical risk stratification. While platelet count increases below the threshold significantly improve prognosis, further increases above this threshold may elevate mortality risk. Future prospective studies should validate these findings in diverse populations and investigate underlying mechanisms.

## Data Availability

The original contributions presented in the study are included in the article/Supplementary material, further inquiries can be directed to the corresponding author.
