# Supplementary material for: Association between platelet count and 30-day in-hospital mortality among intensive care unit patients with sepsis: a multicenter retrospective cohort study
Source: Front Med (Lausanne). 2025 Jan 20;11:1444481. doi: 10.3389/fmed.2024.1444481 (PMC11788309; doi:10.3389/fmed.2024.1444481)
Supplement: Supplementary file 1 [file Table_1.DOCX]

**Association between platelet count and 30-day in-hospital mortality among intensive care unit patients with sepsis: a multicenter retrospective cohort study**

**Running head**: The relationship between platelet and 30-day in-hospital mortality

Jun Wang^1#^, Pan Zhou^2#^, Xin Li^2#^, Li Zhou^2^, Zhe Deng^2*^

^#^Jun Wang, Pan Zhou and Xin Li contributed equally to this work and are acknowledged as the co-first authors.

^*^**Correspondence to**: Zhe Deng (dengz163@163.com)

**Author’s affiliation:**

^1^ Department of Intensive Care Unit, Shenzhen Baoan ShiYan People's Hospital.

^2^Department of Emergency Medicine, the First Affiliated Hospital of Shenzhen University, Shenzhen Second People’s Hospital, Shenzhen, 518035, China.

**Conflict of interest:** None.

**Table S1. Collinearity diagnostics steps**

| **Variable** | **VIF** |
| --- | --- |
|  | **Step 1** |
| Sex | 1.0 |
| Ethnicity | 1.0 |
| Age(years) | 1.2 |
| Hb(g/dl) | 1.3 |
| BUN((mg/dl)) | 1.8 |
| Scr(mg/dl) | 1.7 |
| ALB(g/dl) | 1.3 |
| APACHE-Ⅳ score | 1.4 |
| ARF | 1.3 |
| AF | 1.1 |
| ACS | 1.1 |
| CHF | 1.1 |
| COPD | 1.1 |
| stroke | 1.0 |
| Diabetes mellitus | 1.1 |
| Hypertension | 1.1 |
| Antiplatelet | 1.1 |
| Glucocorticoid | 1.1 |
| Carbapenems | 1.1 |
| Cephalosporins | 1.2 |
| Levofloxacin | 1.1 |
| Vancomycin | 1.3 |
| Mechanical ventilation | 1.4 |

Abbreviation: VIF: variance inflation factor; VIF = 1/ (1-R^2^).

Note: The variables with VIF>5 will be regarded as collinear variables and cannot be included in the multiple regression model

Hb, hemoglobin; BUN, blood urea nitrogen, Scr, creatinine; ALB, albumin; APACHE-IV scores, Acute Physiology and Chronic Health Evaluation-IV score; ARF, acute respiratory failure; AF, atrial fibrillation; ACS, acute coronary syndrome; CHF, congestive heart failure; COPD, chronic obstructive pulmonary disease.

**Table S2. Influencing factors of 30-day in-hospital mortality using univariate regression analysis**

| **Variable** | **Statistic** | **OR95%CI** | ***P*** |
| --- | --- | --- | --- |
| Sex |  |  |  |
| male | 9401 (52.3%) | 1.0 |  |
| Female | 8576 (47.7%) | 0.97 (0.89, 1.04) | 0.3881 |
| Ethnicity |  |  |  |
| Caucasian | 13810 (76.82%) | 1.0 |  |
| African American | 1799 (10.01%) | 0.99 (0.87, 1.13) | 0.8647 |
| Hispanic | 861 (4.79%) | 1.20 (1.01, 1.43) | 0.0427 |
| Asian | 377 (2.10%) | 0.88 (0.66, 1.17) | 0.3794 |
| Other/unknown | 1130 (6.29%) | 1.02 (0.87, 1.20) | 0.8331 |
| Age(years) | 65.26 ± 16.20 | 1.02 (1.02, 1.02) | <0.0001 |
| Hb(g/dl) | 11.38 ± 2.54 | 0.93 (0.92, 0.95) | <0.0001 |
| Platelet count (×10^9^/l) | 226.8 ± 114.6 | 0.98 (0.98, 0.99) | <0.0001 |
| BUN (mg/dl) | 34.78 ± 26.67 | 1.01 (1.01, 1.01) | <0.0001 |
| Scr(mg/dl) | 1.98 ± 1.95 | 1.04 (1.02, 1.06) | <0.0001 |
| ALB(g/dl) | 2.86 ± 0.74 | 0.54 (0.51, 0.57) | <0.0001 |
| APACHE-Ⅳ score | 70.46 ± 26.92 | 1.03 (1.03, 1.03) | <0.0001 |
| **Comorbid conditions** |  |  |  |
| ARF |  |  |  |
| No | 12034 (66.94%) | 1.0 |  |
| Yes | 5943 (33.06%) | 2.24 (2.07, 2.42) | <0.0001 |
| AF |  |  |  |
| No | 15438 (85.88%) | 1.0 |  |
| Yes | 2539 (14.12%) | 1.77 (1.60, 1.96) | <0.0001 |
| ACS |  |  |  |
| No | 16681 (92.79%) | 1.0 |  |
| Yes | 1296 (7.21%) | 1.71 (1.50, 1.95) | <0.0001 |
| CHF |  |  |  |
| No | 16002 (89.01%) | 1.0 |  |
| Yes | 1975 (10.99%) | 1.37 (1.22, 1.54) | <0.0001 |
| COPD |  |  |  |
| No | 16094 (89.53%) | 1.0 |  |
| Yes | 1883 (10.47%) | 1.09 (0.96, 1.23) | 0.1882 |
| stroke |  |  |  |
| No | 17449 (97.06%) | 1.0 |  |
| Yes | 528 (2.94%) | 2.00 (1.65, 2.42) | <0.0001 |
| Diabetes mellitus |  |  |  |
| No | 15099 (83.99%) | 1.0 |  |
| Yes | 2878 (16.01%) | 0.96 (0.86, 1.07) | 0.4423 |
| Hypertension |  |  |  |
| No | 16000 (89.00%) | 1.0 |  |
| Yes | 1977 (11.00%) | 0.93 (0.82, 1.06) | 0.2899 |
| **Treatment** |  |  |  |
| Antiplatelet |  |  |  |
| No | 16993 (94.53%) | 1.0 |  |
| Yes | 984 (5.47%) | 1.11 (0.94, 1.31) | 0.2069 |
| Glucocorticoid |  |  |  |
| No | 16120 (89.67%) | 1.0 |  |
| Yes | 1857 (10.33%) | 1.21 (1.07, 1.37) | 0.0023 |
| Carbapenems |  |  |  |
| No | 17263 (96.03%) | 1.0 |  |
| Yes | 714 (3.97%) | 1.33 (1.11, 1.60) | 0.0022 |
| Cephalosporins |  |  |  |
| No | 15993 (88.96%) | 1.0 |  |
| Yes | 1984 (11.04%) | 0.87 (0.76, 0.99) | 0.0318 |
| Levofloxacin |  |  |  |
| No | 16674 (92.75%) | 1.0 |  |
| Yes | 1303 (7.25%) | 1.06 (0.92, 1.23) | 0.4233 |
| Vancomycin |  |  |  |
| No | 14704 (81.79%) | 1.0 |  |
| Yes | 3273 (18.21%) | 1.20 (1.09, 1.33) | 0.0002 |
| Mechanical ventilation |  |  |  |
| No | 11796 (65.62%) | 1.0 |  |
| Yes | 6181 (34.38%) | 2.93 (2.70, 3.17) | <0.0001 |

Hb, hemoglobin; BUN, blood urea nitrogen, Scr, creatinine; ALB, albumin; APACHE-IV scores, Acute Physiology and Chronic Health Evaluation-IV score; ARF, acute respiratory failure; AF, atrial fibrillation; ACS, acute coronary syndrome; CHF, congestive heart failure; COPD, chronic obstructive pulmonary disease.
